# A Consideration of Some of the Causes of Dental Decay

**Published:** 1883-08

**Authors:** Alfred Carpenter


ARTICLE V.

### A CONSIDERATION OF SOME OF THE CAUSES WHICH GIVE RISE TO DENTAL DECAY.—(Continued.)

BY ALFRED CARPENTER, M. D.

(Transactions of Odontological Society, published in London *Record.*)

Future history will show whether the children of total abstainers have an advantage over those of non-abstainers as regards soundness of teeth, at present, there is no proof that the acknowledged benefit of total abstinence does give any immunity as regards hereditary dental disease, but that abstinence does assist to save the teeth of the gouty inclined, I have had too many instances before me to doubt the fact, whilst the sufferings which stimulants induce in those who indulge in them is also as manifest. Celsus writes in his sixth book, " De Medicina "—*In dentium autem dolore qui ipse quoque maximis tormentis annumerari potest, vinum ex toto circumcidendum est. Cibo quoque primo abstenandum.*" That ancient and generally accurate writer is very decided upon this point, and yet it is curious how some modern physicians ignore the teaching of that acute observer, and prescribe alcohol in all its forms, and in excessive doses, for the relief of dental neuralgia, with the moral certainty that they are only postponing the evil day, and adding to its ultimate intensity. The paralysis which alcohol induces in the vessels supplying the sentient nerve gives temporary relief, but the pain of to-morrow is all the greater from the anæsthesia of to-day. Of two patients, suffering from dental caries, and who are intent upon continuing the presence of the offending organ in the mouth, the one who submits to the pain without using anæsthetic remedies will get over the action which is causing the neuralgia much sooner, and will get earlier relief, with a far less expenditure of patience in suffering, than the man or woman who flies to stimulants for temporary relief from their distress. The alcohol simply delays the action which is necessary for the removal of the exciting cause, and does not at all assist to its cure. In theorizing however, upon the subject of alcoholic indulgence, we must bear in mind that, as a rule, children are total abstainers, but I believe that, if we could separate those who suffer from hereditary causes from those who have no hereditary taint in their dental appendages, and then follow the total abstainers through life, and compare them in their decline of years, I think it would be found that the total abstainers had suffered comparatively little from neuralgia, or any painful malady in the dental organs, as compared with the wine-bibber.

I do not yet know enough of the alliance between dental diseases and rheumatism, or of the connection, if any, with the cancerous cachexia, to make any specific allusion to those tendencies, even if time would allow me to do so; but there is a field for observation in connection with the development of lactic acid in the constitution, and the rheumatic diathesis, which the state of the teeth might assist to determine. I leave that for others to follow out. If I have given lines for the future thought to those who have a more immediate connection with the results of dental disease than I have, I shall have effected my object. I think that our design should be, not the establishment of a foregone conclusion by bending our facts to our theories, but a steady observance of facts, a correct registration of conditions, and then in the end a proper generalism may be obtained, which will be based upon the truth. This should be the desire of every one of us, to get established upon a sound and satisfactory line, even if its establishment should lead to a large diminution of that work, which has already taken place in hospital practice, and made conservative surgery of more importance than heroic performance. It will materially diminish extractions, and render prevention of disease of great import, even to the surgeon-dentist, than its radical cure can ever be.

